# Born to be bee, fed to be worker? The caste system of a primitively eusocial insect

**DOI:** 10.1186/1742-9994-9-35

**Published:** 2012-12-10

**Authors:** Nayuta Brand, Michel Chapuisat

**Affiliations:** 1 Department of Ecology and Evolution, University of Lausanne, Lausanne 1015, Switzerland

**Keywords:** Evolution of eusociality, Caste differentiation, Parental manipulation, Provisioning behaviour, Sweat bees, Halictids, *Halictus scabiosae*

## Abstract

**Introduction:**

Primitively eusocial halictid bees are excellent systems to study the origin of eusociality, because all individuals have retained the ancestral ability to breed independently. In the sweat bee *Halictus scabiosae*, foundresses overwinter, establish nests and rear a first brood by mass-provisioning each offspring with pollen and nectar. The mothers may thus manipulate the phenotype of their offspring by restricting their food provisions. The first brood females generally help their mother to rear a second brood of males and gynes that become foundresses. However, the first brood females may also reproduce in their maternal or in other nests, or possibly enter early diapause. Here, we examined if the behavioural specialization of the first and second brood females was associated with between-brood differences in body size, energetic reserves and pollen provisions.

**Results:**

The patterns of variation in adult body size, weight, fat content and food provisioned to the first and second brood indicate that *H. scabiosae* has dimorphic females. The first-brood females were significantly smaller, lighter and had lower fat reserves than the second-brood females and foundresses. The first-brood females were also less variable in size and fat content, and developed on homogeneously smaller pollen provisions. Foundresses were larger than gynes of the previous year, suggesting that small females were less likely to survive the winter.

**Conclusions:**

The marked size dimorphism between females produced in the first and second brood and the consistently smaller pollen provisions provided to the first brood suggest that the first brood females are channelled into a helper role during their pre-imaginal development. As a large body size is needed for successful hibernation, the mother may promote helping in her first brood offspring by restricting their food provisions. This pattern supports the hypothesis that parental manipulation may contribute to promote worker behaviour in primitively eusocial halictids.

## Introduction

The hallmark of eusociality is reproductive division of labour between generations, a surprising social organization by which some individuals become functionally sterile helpers [1]. Primitively eusocial species are excellent systems to study the proximate mechanisms and ultimate causes leading to eusociality, because helpers have retained the ancestral ability to breed independently and may thus obtain both direct fitness benefits through reproduction and indirect fitness benefits by helping relatives [2-4].

Primitively eusocial halictids have a low degree of morphological differentiation between queens and helpers and a high degree of behavioural flexibility in both types of individuals [2]. As a result, females have multiple reproductive options that result in diverse types of social organisation. Many primitively eusocial species live in temperate zones, where females overwinter, found nests either alone or in association, and raise two broods per year [2]. The first brood daughters may become non-reproductive helpers that stay in their natal nest to assist their mother in raising a next brood of gynes and males. However, they may also gain direct fitness by reproducing in their natal nest, drifting to reproduce in other nests, or entering early diapause to become nest foundresses in the next spring [5-10].

In eusocial halictids, the various reproductive strategies of females are generally associated with some difference in body size [2,11,12]. Foundresses tend to be large-bodied females that have large energetic reserves enabling them to overwinter, establish nests and reproduce independently [13]. In contrast, helpers tend to be smaller-bodied daughters. For instance, across eight halictid species the proportion of helpers with undeveloped ovaries correlated with the degree of size divergence between foundresses and helpers [14]. Body size depends in part on larval diet, which has long been recognized to play an important role in caste differentiation and sociality [15]. For example, in primitively eusocial *Polistinae* wasps it has been proposed that the castes result from differential nourishment during larval development, with individuals experiencing relatively poor diet tending to become workers [16-18].

An interesting aspect of body size variation and diet is that that the mother might limit the amount of resources that she provides to her offspring, thus forcing them to develop into small and lean females that are incapable of independent reproduction and are thus constrained to become helpers [19-24]. Moreover, small-bodied females may be easier to manipulate into a subordinate role by dominance interactions and aggression [19,25]. In line with the hypothesis of parental manipulation, in *Polistes metricus* hand-fed female larvae became heavier and were more cold-resistant than those fed only by the queen [26].

Maternal control of body size is likely to be particularly effective in mass-provisioning species such as social halictids, which lay a single egg on a mass of pollen and nectar deposited in a closed cell, thus providing all the food that the offspring will need to develop into adulthood. In all annual species of eusocial sweat bees studied so far, pollen provisions of gyne-destined larvae were larger than those of worker-destined larvae [11,27]. Moreover, in one species the provisions provided to female offspring were more variable than the ones provided to male offspring [28]. Overall, in various bee species the provision quality and quantity were shown to affect adult body size, as well as the sex of the egg laid [29-32]. Together, these data indicate that mothers can control the body size of their offspring in mass-provisioning bees. It is therefore of interest to study the relationship between pollen provisions, body size and behaviour in species that have complex social systems, and where the first generation of offspring have multiple options.

Body size variation also provides insights into the ecology, reproductive strategy and social behaviour of a species. If all offspring have similar fitness functions, a simple model predicts that there is a single optimal amount of resource that a parent should expend on each offspring [33]. Therefore, variations in parental expenditure and offspring body size generally reflect changes in availability of the limiting resources, in fitness expectations or in offspring role, for example switch from reproducing to helping [28,34].

Here, we study body size variation in the sweat bee *Halictus scabiosae* (Rossi, 1790), a ground-nesting, mass-provisioning halictid that varies in social organization and relatedness among nestmates [10,35]. Our objectives are to examine if the mothers restrict offspring resources to promote worker behaviour in their first brood and to document the degree of body size differences between broods, which may contribute to explain behavioural specialization. In Switzerland, *H. scabiosae* forms annual colonies in which females raise two broods that are well-separated in time [10,35]. In spring, the foundresses – mated females that have overwintered – found new colonies, either alone or in small groups [10]. The foundresses raise a first brood (B1) that is female-biased and emerge from the nests in June and July [10]. Many of the B1 females do not reproduce and help their mother to raise a second brood (B2) of females and males [10,35,36]. However, the B1 females have retained the ability to mate and lay eggs, so they have the possibility to reproduce in their natal nest or in neighbour nests [10]. The B2 females and males emerge from the nest in August and September. After mating, the B2 females enter diapause to pass the winter and become the next spring foundresses. Whether some of the B1 females enter early diapause, overwinter and found new colonies in the next spring, as has been documented in another species [37], remains to be investigated.

An interesting aspect of *H. scabiosae* is that queen turnover and drifting occur frequently, leading to low average relatedness between foundresses, B1 and B2 females [10, Brand and Chapuisat, unpublished data]. The fact that B1 helpers often raise unrelated brood could limit the benefits of size manipulation by foundresses and select for large-sized B1 females, if large B1 females have a higher probability to become replacement queens or found new nests.

Early reports on the degree of body size dimorphism between *H. scabiosae* foundresses and helpers (= B1 females) are somewhat equivocal, in part because of small sample sizes and variation in measurement methods. A single foundress was reported to be larger than three of her helpers [38]. When measuring wing and abdomen length in a larger sample of bees, Knerer [39] documented that foundresses were on average larger than helpers, but with a continuous distribution and a large overlap of sizes. In contrast, Batra [35] found no size difference between foundresses and helpers when measuring the head width of 30 bees from seven nests. Hence, more data on body size variation among female types (foundresses, B1 and B2 offspring) are needed to better understand the social organisation, partitioning of reproduction and reproductive options in this primitively eusocial sweat bee.

In this study, we compared adult body size, weight and fat content of foundresses, first brood females and second brood females in *H. scabiosae*. We also compared the pollen and nectar provisions provided to the first and second brood, in order to evaluate if the mothers might influence the body size of their first brood offspring by limiting their food resources. Finally, we examined if body size was correlated with the probability to survive the winter. These data on the degree, origin and consequence of dimorphism between breeders and helpers will help to evaluate if parental manipulation influences body size and helping in social groups with low relatedness.

## Results

### Caste differentiation

**Figure 1:**
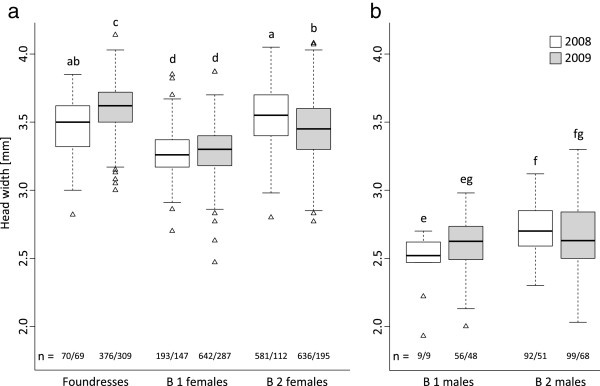
**Head size of *H. scabiosae *bees sampled in 2008 (white bars) and 2009 (grey bars).** (**a**) Foundresses, first brood females and second brood females. (**b**) First brood males and second brood males. Solid lines indicate the median for each category, boxes the interquartile range, and whiskers the most extreme values within 1.5 times the interquartile range. Sample sizes for each category (number of individuals/number of nests) are indicated above the x-axis. Different letters indicate significant differences between groups (Tukey's tests). Females and males were analysed separately.

The analysis of 2498 bees from 769 nests revealed that the foundresses, first brood (B1) females and second brood (B2) females differed significantly in head width (Figure 1a; effect of female type: log-likelihood ratio, LR = 11.00, *P* < 0.01). In both years, the B1 females were significantly smaller than both the B2 females (Figure 1a; Tukey's tests: 2008, |z| =17.51, *P* < 0.001; 2009, |z| = 13.33, *P* < 0.001) and foundresses (Tukey's tests: 2008, |z| = 7.81, *P* < 0.001; 2009, |z| = 26.55, *P* < 0.001). Within the same nests, the degree of head size dimorphism between foundresses and B1 females (calculated as follows: [(foundress head width - B1 female head width) / foundress head width]) was 0.09 ± 0.07 (n = 111 nests), while the size dimorphism between B2 and B1 females was 0.05 ± 0.07 (n = 209 nests). The variance in head width also differed significantly among female types (heteroscedasticity: LR = 63.90, *P* < 0.001): the B1 females were the least variable, with the variances in head width being 1.8 and 1.3 times larger in B2 females and foundresses, respectively (Figure 1a).

Nest identity had a significant effect on head width (LR = 59.82, *P* < 0.001). The year had no main effect on head width (LR = 9.37e-7, *P* = 0.99), but there was a significant interaction between female type and year (LR = 59.91, *P* < 0.001). This is because the B2 females were significantly smaller than the foundresses in 2009 (Tukey's test: |z| = 12.76, *P* < 0.001), but not in 2008 (Tukey's test: |z| = 2.63, *P* = 0.08; Figure 1a). The B2 females of 2008 were also significantly smaller than the foundresses of 2009 (Tukey's test: |z| = 4.91, *P* < 0.001), which indicates that within this cohort the larger females were more likely to survive the winter.

The foundresses, B1 females and B2 females sampled in 2009 differed significantly in dry weight (Table 1; effect of female type: LR = 19.49, *P* < 0.001). In line with their smaller head size, the B1 females had a significantly lower dry weight than both the B2 females (Tukey's test: |z| = 3.99, *P* < 0.001) and foundresses (Tukey's test: |z| = 4.16, *P* < 0.001). In contrast, the weight of the B2 females was not significantly different from the one of the foundresses (Tukey's test: |z| = 0.37, *P* = 0.93).

**Table 1:** Adult dry weight and fat weight (2009)

	**n**	**Dry weight**	**Fat weight**	**Proportion of fat**
		**(mg ± SD)**	**(mg ± SD)**	
Foundresses	28	26.13 ± 4.26	1.81 ± 0.89	6.9 ± 4.1 %
First brood females	23	20.93 ± 3.80	1.23 ± 0.41	5.8 ± 3.5 %
Second brood females	34	25.94 ± 5.94	2.52 ± 1.72	9.2 ± 4.6 %
Second brood males	13	13.16 ± 4.34	0.79 ± 0.60	5.5 ± 3.9 %

n = number of nests.

The three female types also differed significantly in absolute fat weight (Table 1; effect of female type: LR = 15.49, *P* < 0.001). Again, the B1 females had a lower absolute fat weight than both the B2 females (pairwise Wilcoxon tests, W = 546.5, *P* = 0.01) and foundresses (W = 194.5, *P* = 0.02). The B2 females and foundresses did not differ significantly in fat weight (W = 546.5, *P* = 0.32). The variances in fat weight were significantly different for foundresses, B1 and B2 females (heteroscedasticity: LR = 19.08, *P* < 0.001): the B1 females were the least variable in fat weight, while the B2 females and foundresses had 5.2 and 1.8 times larger variances in fat weight, respectively.

**Figure 2:**
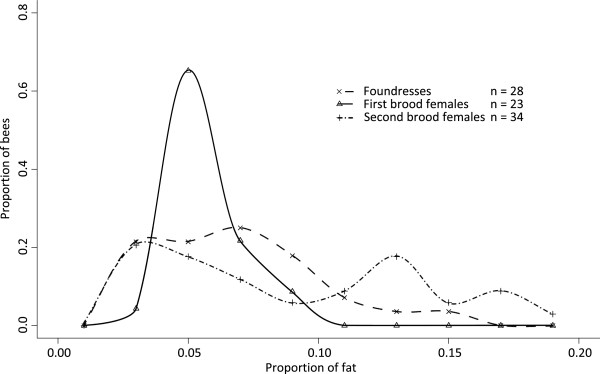
**Relative fat content of female bees from 2009.** Distribution of bees according to their proportion of fat over total dry weight for foundresses (dashed line), first brood females (solid line) and second brood females (point-dashed line). n = number of nests.

The relative fat content (fat weight divided by total dry weight) did not differ significantly between foundresses, B1 and B2 females (effect of female type: LR = 7.53, *P* = 0.11), and was not explained by female head width (LR = 1.28, *P* = 0.73) nor by an interaction between female type and head width (LR = 0.77, *P* = 0.68). However, the variances in relative fat content were significantly different for foundresses, B1 and B2 females (heteroscedasticity: LR = 22.82, *P* < 0.001; Figure 2). Again, the B1 females were the least variable, and the variances in relative fat content of the B2 females and foundresses were 7.0 and 3.0 times larger, respectively. The relative fat content of the B2 females appeared to be multimodal: about 56% (19 of 34) of the B2 females had a low fat content (median at 5.3%), similar to the one of the B1 females, while the rest of the females had a larger fat content (median at 16%; Figure 2).

The males from the first and second brood differed significantly in head width (Figure 1b; effect of brood: LR = 6.94, *P* < 0.01). However, the difference appeared to be small and not consistent across years (Figure 1b). The B1 males were significantly smaller than the B2 males in 2008 (Tukey's test: |z| = 3.31, *P* < 0.01), but not in 2009 (Tukey's test: |z| = 2.06, *P* = 0.15), when we sampled a much larger number of B1 males (Figure 1b). The variance in head width did not differ significantly between B1 and B2 males (heteroscedasticity: LR = 0.32, *P* = 0.57).

The males had a significantly smaller head width than the females (Figure 1a and b; effect of sex: LR = 29.62, *P* < 0.001). There was again a significant effect of nest identity on male and female head width (LR = 98.9, *P* < 0.001). The males were also significantly lighter than the females in terms of dry weight (Table 1; *t*-test: t = 6.45, df = 13.30, *P* < 0.001), fat weight (Table 1; Wilcoxon-test: W = 940, *P* < 0.001) and relative fat content (W = 741, *P* = 0.049).

### Brood provisions

We sampled pollen and nectar provisions in 2009. The provisions provided to the first brood were significantly smaller than the ones provided to the second brood (Table 2; fresh weight: LR = 16.76 *P* < 0.001; dry weight: LR = 16.86, *P* < 0.001). The variance in weight was 3.6 (fresh weight) and 3.3 (dry weight) times larger for provisions of B2 offspring than for the ones of B1 offspring (heteroscedasticity: fresh weight: LR = 5.71, *P* = 0.02; dry weight: LR = 5.04, *P* = 0.02). The provisions provided to B2 offspring contained slightly more sugar (17.5% in weight) than the ones provided to B1 offspring, but this difference was not significant (Table 2; LR = 2.55, *P* = 0.11). The proportion of sugar (sugar weight divided by total dry weight) was on average higher and more variable in provisions of B1 offspring than in the ones of B2 offspring (Table 2; effect of brood: LR = 6.8, *P* = 0.01; heteroscedasticity: LR = 12.21, *P* < 0.001).

**Table 2:** Pollen provisions provided to the first and second brood (2009)

	**n**	**Fresh weight**	**Dry weight**	**Sugar weight**	**Proportion of sugar**
		**(mg ± SD)**	**(mg ± SD)**	**(mg ± SD)**	
First brood	16	125.5 ± 19.9	77.5 ± 12.9	34.3 ± 11.3	43.6 ± 8.4 %
Second brood	16	177.4 ± 37.5	112.3 ± 23.4	40.3 ± 9.4	36.0 ± 4.0 %

n = number of pollen balls.

## Discussion

The females of *H. scabiosae* were clearly dimorphic. On average, females originating from the first brood (B1) were significantly smaller, lighter and had lower absolute fat reserves than both foundresses and females produced in the second brood (B2). The relative mean size difference between the B2 and B1 females at the population level amounted to 6%, 24% and 105% for head width, dry weight and fat weight, respectively.

In insects, the head size of adults doesn't change after the cuticle of the head capsule has fully sclerotized. Adult head size generally depends on the genotype and on food quality and quantity during development [29]. These factors are likely to vary among colonies, which is in line with the finding that nest identity had a significant effect on head width in our and other studies [29,40]. The degree of head size dimorphism between B2 and B1 females within nests of *H. scabiosae* (5%) was slightly lower than the one recorded in other socially polymorphic and weakly eusocial halictids, such *H. sexcinctus* (7.5%), *H. ligatus* (8%) and *H. poeyi* (10%) [27,40-42]. Moderate dimorphism in *H. scabiosae* is consistent with the finding that females have flexible reproductive strategies [10]. Interestingly, in *H. rubicundus* the degree of wing length dimorphism between foundresses and B1 females was very low (0.3%) compared to wing length dimorphism between B2 and B1 females (4.3%)*,* because many of the foundresses were B1 females that had overwintered [5,37]. In comparison, the high degree of head size dimorphism between foundresses and B1 females (9%) suggests that most B1 females do not over-winter in our study population of *H. scabiosae*.

Within each category of females (foundresses, B1 and B2), the head width, dry weight and fat weight showed a large amount of variation, and the size distributions of the three categories partially overlapped. Size variation may reflect changes in the number of foragers [28], in resource availability [40], or in parental allocation. Importantly, the variance in head width and fat weight was significantly and consistently larger in B2 females (gynes) and foundresses than in B1 females (workers), even after controlling for differences in means. The reverse pattern was found in advanced eusocial insects: the variance in size was greater for workers than for queens in formicine ants and vespine wasps [43,44], suggesting lower selection pressure on castes that are no longer capable of direct reproduction [44]. In contrast, the low size variability in B1 females of *H. scabiosae* is consistent with the parental manipulation hypothesis [22]: it suggests that foundresses constrain the food resources to rear uniformly small B1 females that will behave as workers. Conversely, if the survival and fecundity of reproductive females (gynes) increase gradually with body size and energetic reserves [13,29,45], variation in resources or brood number might result in high size variability in B2 females.

The pollen and nectar provisions provided to the B2 offspring were much larger and more variable in size than the ones provided to the B1 offspring. The difference amounted to 45% in terms of dry weight. Such differential provisioning of the first and second brood has been documented in several eusocial halictine bees [11,27,46]. It would be interesting to investigate provisioning in species that are facultatively social [47], as well as in eusocial and parasocial colonies exhibiting split sex-ratio [48]. Somewhat surprisingly, in *H. scabiosae* there was no significant difference between B1-destined and B2-destined provisions in terms of total sugar weight, due to the higher average sugar concentration in spring provisions. Our study is the first to find that provisions fed to B1 offspring have a higher but more variable concentration of sugar. In contrast, in *H. ligatus* the sugar concentration was higher in gyne-destined than in male-destined and B1 female-destined provisions [27]. Variation in sugar content may reflect differences in sex ratio, variation in the number of foragers [28], or temporal and seasonal variation in nectar quality and availability, for example due to weather conditions [27,40].

The smaller pollen provisions provided to the first brood are consistent with the idea of parental manipulation [22]. Indeed the foundresses may force their first offspring to behave as helpers by restricting their food provisions in such a way that they become small, lean females unable to establish independent colonies, particularly if large energetic reserves are needed to survive the winter or to nest independently [13,19,23,27]. It is somewhat surprising to find signs of parental manipulation in a species that has high rates of queen turnover and high incidence of drifting, which leads to a low relatedness between foundresses, B1 and B2 females in part of the nests [10, Brand and Chapuisat, unpublished data]. If colony relatedness becomes very low, the B1 females should be selected to resist manipulation and claim their share of reproduction [20,21].

In *H. scabiosae*, first brood females occasionally replace foundresses in orphaned nests, forming semisocial colonies [10, Brand and Chapuisat unpublished data]. Overall, the first females appear to be sufficiently large to become replacement queens in existing colonies, but to lack the energetic reserves that are necessary for independent colony founding and overwintering [13].

Parental manipulation is hard to distinguish from seasonal variation in resource availability and resource acquisition, which are influenced by vegetation, weather, photoperiod, number of colony members foraging [28], as well as parasitism and predation risks [49]. Annual weather variation appeared to have had some impact on body size in our population, since B2 females were smaller in 2009, a year with frequent rainfalls during the period of B2 provisioning (late June to mid July). Similarly, foundresses were larger in 2009, after a harsh winter with a temperature drop towards the end of hibernation (late February). Interestingly, B1 female size was very similar over the two years despite pronounced differences in weather conditions in spring, which is consistent with the hypothesis that the mothers control and restrict the provisions destined to the B1 offspring.

Foundresses had significantly larger head size than gynes of the previous year, which suggests that small females were less likely to survive the winter. A similar pattern has been documented in *Bombus terrestris* introduced to Japan [50]. As *H. scabiosae* has expanded its range to the north in recent years [51], it is possible that the body size of gynes is not yet adapted to the winter of Switzerland. More importantly, the higher size of foundresses sampled in spring than gynes sampled in the previous autumn, combined with the small size of first brood females, suggest that first brood females are unlikely to survive the winter. The relative fat content of gynes (9.4%) was surprisingly low compared to other studies (e.g. *H. ligatus,* 17.8%) [13,27]. It seems likely that gynes continue to build up fat stores after their first exit from the nest. This may contribute to explain the low difference in fat weight and fat content between gynes and foundresses that have overwintered, along with the fact that the foundresses were caught at an early stage of colony founding [13].

The males were smaller than females, as commonly observed in insects [29,45]. They also had very low overall fat content and a proportion of fat comparable to the one of B1 females, consistent with the idea that fat reserves are for overwintering and colony founding [13]. In contrast to females, males from the first and second brood showed no clear and consistent differences in size and size variances, which is the expected pattern if variation in female size is due to parental manipulation rather than environmental variation [28]. This result should however be interpreted with caution, because sample sizes were smaller for males than for females.

## Conclusion

The marked size dimorphism between females produced in the first and second brood and the consistently smaller pollen provisions provided to the first brood suggest that the first brood females of the sweat bee *H. scabiosae* are channelled into a helper role during their pre-imaginal development. As a large body size is needed for successful hibernation, the mother may promote helping in her first brood offspring by restricting their food provisions. This pattern, which is common to many primitively eusocial halictids, supports the hypothesis that worker behaviour is in part enforced by parental manipulation of the brood resources in mass-provisioning bees.

## Methods

### Sampling and measurement of bees

Our study site is located in Adlikon, near Zürich, in northern Switzerland. It consists of a dry, south-exposed and sparsely vegetated embankment. *H. scabiosae* is abundant at this site, with more than 1000 nests per breeding season over an area of ca. 30 x 10 meters. We marked nests with numbered nails and flags. We captured the bees by posing net traps on the nest entrance in the early morning (6–8 am), before the bees became active (8:30–10 am). We sampled most foundresses in May and June, most adult bees originating from the first brood (B1) in July, and all adult bees from the second brood (B2) in August and early September. In spring, we detected multiple foundress associations in 16% of the nests, but the vast majority of these associations appeared to be transient and were not resampled later in the season. As the season progressed, bees from earlier cohorts (foundresses or B1 females) could easily be distinguished by the wear of their wings, mandibles and hairs [52].

Head width is commonly used as a proxy for overall adult body size in halictid bees and other insects [40,53,54]. In 2008 and 2009, we measured the head width of 2754 live bees originating from 791 nests. We briefly immobilized the bee on a sponge and measured its largest head width across the eyes, using a precision calliper (SPI 2000 dial calliper, SPI, CA). To diminish measurement errors, we measured each bee three times and used the mean value for subsequent analysis. The coefficient of variation across the three measures was 0.01. To avoid double measurements, we marked the bees on the thorax with a dot of honeybee-marking enamel paint (Apicolori, Bienen-Meier Künten) before releasing them.

In 2009, we measured the weight and fat content of a sub-sample of 109 adult bees originating from 98 nests. At the start of the period of activity of bees, between May 17th and June 6th, we captured 28 foundresses from 28 nests. Later in the season, we captured first brood females (25 individuals from 23 nests, June 25–29), second brood females (41 individuals from 34 nests, August 11–31) and second brood males (15 individuals from 13 nests, August 11–September 8). For the weight analysis, we used bees that were captured upon their first exit from the nest*.* We froze the bees, dried them for five days at 65°C, and measured their dry weight with a microbalance (Mettler Toledo MT5). To measure their fat content, we extracted the lipids by soaking the bees in petroleum ether for 10 days, replacing the ether once. After this extraction, we dried the bees again, re-weighed them, and estimated their fat weight as the dry weight loss between the two measures.

### Brood provisions

To compare the provisions provided to B1 and B2 offspring, we excavated nests and collected the contents of brood cells. In the early morning, we humidified the soil around nest entrances and blew starch into the burrows to follow them more easily while digging. A complete provision consisted of an intact ball of pollen and nectar, enclosed in a sealed brood cell containing a bee egg. On May 21st, 2009, we collected 16 complete provisions prepared for B1 offspring, from eight nests. Between July 22nd and August 11th, 2009, we collected 16 complete provisions prepared for B2 offspring, from nine nests. These provisions were frozen until further analysis. We could not get any information on the ploidy of the collected eggs.

We measured the fresh weight, dry weight (after 48 h at 65°C) and sugar content of complete provisions. We estimated the sugar content by refractometry, using the method described by Richards and Packer [27] and Kapheim *et al.*[28]. In short, we re-suspended the provisions in 200 μl of H_2_O, estimated the sugar concentration in Brix degrees using a refractometer (Abbe-Refraktometer B, Zeiss, Germany), and converted this into total sugar weight per provision, measured in sucrose equivalents.

To estimate annual weather variation, we used data from the weather station Aadorf/Tänikon, available at http://www.meteoswiss.admin.ch/web/en/services/data_portal.html.

### Statistical analysis

We investigated size differences among female types with linear mixed models (LMM, see [55] for a review). We used stepwise log-likelihood tests and controlled for heteroscedasticity between categories by estimating the variance of the residuals modelled as a linear function of the predictor variables [28,55,56]. This approach permits us to compare variances after controlling for differences in means [28]. To test for size differences between foundresses, B1 and B2 females, we included the female type as a fixed effect in the model. In order to control for the non-independence of bees sampled from the same nests and for the effect of the year, we also included the nest identity and year of sampling as random effects. We used Tukey's HSD post-hoc tests to examine which type of female differed from one another. We used similar models to examine size differences between male broods and between sexes.

To examine variation among adult bees in dry weight, fat weight and relative fat content (fat weight divided by total dry weight), we included the type of female or the sex as a fixed effect in a generalized least square model (GLS) [55]. For the analysis of the relative fat content of females, we also included head width as a covariate. For these weight data, as we had measured a single bee for most of the nests (90 out of 98), we used one mean value per nest to ensure the independence of the data. We log-transformed the weight data to have randomly distributed residuals.

We used linear mixed-effects models to compare the pollen provisions provided to the first and second brood. We included the brood (B1 or B2) as a fixed effect. To control for the non-independence of pollen balls sampled from the same nest, we included the nest identity as a random effect. All statistical analyses were carried out with the software R 2.14.0 [57] using the R packages nlme 3.1 [58] and multcomp 1.2 [59].

## Competing interests

The authors declare that they have no competing interests.

## Authors’ contributions

NB and MC designed the study. NB performed the field work, chemical analyses and statistical analyses. MC provided guidance and advised on data analysis. NB and MC wrote the manuscript. All authors read and approved the final manuscript.
